# Reversing and modulating cellular senescence in beta cells, a new field of opportunities to treat diabetes

**DOI:** 10.3389/fendo.2023.1217729

**Published:** 2023-09-26

**Authors:** Maria F. Rubin de Celis, Susan Bonner-Weir

**Affiliations:** ^1^ Department of Medicine, Beth Israel Deaconess Medical Center and Harvard Medical School, Boston, MA, United States; ^2^ Joslin Diabetes Center and Harvard Medical School, Boston, MA, United States

**Keywords:** pancreatic beta cells, diabetes, cellular senescence, reverse, senolytics, senomorphics and modulators of cellular senescence

## Abstract

Diabetes constitutes a world-wide pandemic that requires searching for new treatments to halt its progression. Cellular senescence of pancreatic beta cells has been described as a major contributor to development and worsening of diabetes. The concept of reversibility of cellular senescence is critical as is the timing to take actions against this “dormant” senescent state. The reversal of cellular senescence can be considered as rejuvenation of the specific cell if it returns to the original “healthy state” and doesn’t behave aberrantly as seen in some cancer cells. In rodents, treatment with senolytics and senomorphics blunted or prevented disease progression, however their use carry drawbacks. Modulators of cellular senescence is a new area of research that seeks to reverse the senescence. More research in each of these modalities should lead to new treatments to stop diabetes development and progression.

## Introduction

The accumulation of senescent cells is one of the hallmarks of aging, which also include telomere shortening, mitochondrial dysfunction, genomic instability, cell exhaustion, inflammation, abnormal cell to cell communication, fibrosis and microbiome dysregulation (1–4). Cellular senescence is a state of the cell triggered by different intrinsic and extrinsic signals promoted by either stressful conditions or certain physiological conditions (5–7). It has been identified as one of the main triggers for several diseases, and it is being extensively studied.

The phenotype of a senescent cell has been considered as a static and permanent cellular state, in which a cell can no longer proliferate but remains metabolically active, secreting the cytokines, chemokines and other molecules that constitute the senescence-associated secretory phenotype (SASP). Characteristics of a senescent cell include flattened and enlarged shape, heterochromatin foci, lipofuscin granules, DNA scars, and dysfunction in protein processing and gene expression. In addition, senescent cells present increased lysosomal activity (8) and macromolecular damage (2) and are in cell cycle arrest. Even so, the constellation of these characteristics differs with cell type and with the initiating stimulus (2, 7, 9). But, this statement of “irreversibility” has been challenged, and the notion of reversible senescence is getting more support.

As it has been described, cellular senescence is a double-edge sword. Beneficial effects of treatments for cancers, such as therapy-induced senescence (TIS), come from drugs that target specific proteins of the cell cycle machinery to stop proliferation and halt tumor’s growth and metastasis (10–13). However, the accumulation and permanence of senescent cells in tissues can promote tissue dysfunction and aging acceleration, leading to chronic diseases (7, 14–18). Recent evidence that cellular senescence constitutes a major trigger responsible for development and worsening of metabolic disorders, such as diabetes, are opening new avenues to find treatments specifically for these diseases. With the broad options for targeting senescent cells or possibly reversing this state, it is exciting time to find better treatments to halt the progression to diabetes.

In this review, we will focus on the recent findings that support the notion that a major contribution to development and worsening of diabetes is the accumulation of senescent cells in metabolically related tissues and specially in the pancreas (14, 16, 19, 20) and whether the possibility of reversing senescence on beta cells would blunt the disease progression. At the same time as revisiting the concept of irreversibility of cellular senescence, we will review the drugs used in metabolic tissues that have been developed to either remove senescent cells (senolytics) or attenuate the release of the SASP (senomorphics) as well as the potential of the so-called “modulators of cellular senescence” that may reduce the negative effects of senolytics and senomorphics.

## Challenging the “irreversible” dogma of cellular senescence

Cellular senescence was described for the first time by Hayflick and Moorhead in 1961 (21), when they described and characterized the development of 25 strains of human cells from different fetal tissues, finding that normal cells can replicate only for a limited number of passages and then entered in an irreversible cell cycle arrest (6, 21, 22). This leads to the absence of Ki-67 protein, presence of senescence-associated β-galactosidase activity and expression of several tumor suppressors and cell cycle inhibitors. Senescent cells are “arrested” in proliferation, but they remain metabolically active, release the SASP factors (23) and are resistant to apoptosis (24, 25).

Cellular senescence is the response to cellular stress and is promoted by different stressors such as DNA damage, telomere dysfunction, oncogene activation and organelle stress. It has been shown that accumulation of senescent cells in tissues promotes organismal aging and dysfunction.

**Figure 1:**
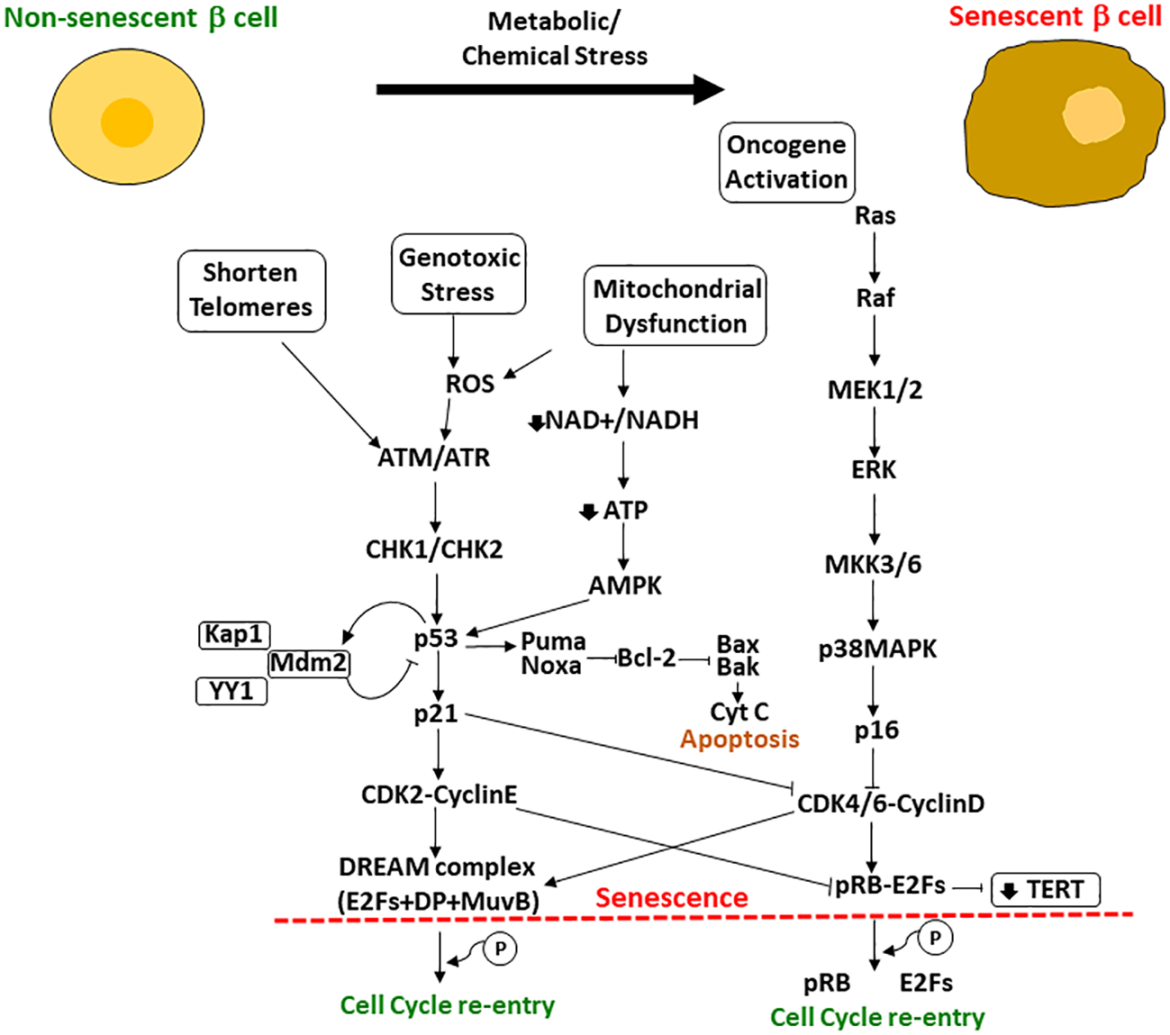
Upstream and downstream effectors of p53/p21^WAF1/CIP1^ and pRB/p16^INK4a^ involved in the regulation of cellular senescence. When cells are exposed to metabolic or chemical stress, the cells suffer changes including shortening of telomeres, mitochondrial dysfunction, genotoxic stress and oncogene activation. Upstream and downstream effectors of p53 and pRB regulate their activation and the resultant cascade leading to cellular senescence or, if possible, cell cycle re-entry. If p53 is high enough, cells undergo apoptosis; if lower p53 levels, the cell can become senescent with upregulation of some antiapoptotic pathways (shown here as Bcl-2) and will survive in cell cycle arrest. Another important effector is the TERT (encodes for the telomerase reverse transcriptase), that is repressed by p21 through the phosphorylated pRB-E2Fs complex CDK2 mediated.

However, several studies have shown that cells can escape the senescent state in certain circumstances. Beausejour et al., 2003 showed that replicative senescence in human fibroblasts could be reversed. In the process of replicative senescence, cells experience a critical shortening of the telomeres, which are a specific DNA sequence plus their associated proteins key to stabilize the ends of the chromosomes (26). However, in this study, they demonstrated that ectopic expression of telomerase to restore telomeres could not protect cells from cellular senescence. In fact, there are other key players in the acquisition of the fully irreversible senescent state that have been identified, such as p53, pRB and their regulatory associated proteins (e.g., cyclin-dependent kinase inhibitor (CDKI) p21 and p16, respectively), constituting the p53/p21^WAF1/CIP1^ and the p16^INK4a^/pRB pathways. Both axes of control of cellular senescence involve upstream and downstream regulators that could serve as targets for treatments to suppress senescent cell accumulation in tissues or for tumor treatments (**Figure 1**). Increased p16 (the regulator of pRB) is key to acquiring an irreversible senescent state. They observed that low levels of p16 contributed to resumption of proliferation upon p53 inactivation and limited growth upon expression of oncogenic RAS. In contrast, cells with high levels of p16 failed to proliferate upon p53 inactivation or RAS expression, even though these cells were able to re-enter the cell cycle after pRb inactivation (26).

P53 expression if high enough results in apoptosis but at a lower level will lead to p21 induced senescence (26–29). However, more studies are needed to elucidate the exact mechanisms and interactions to complete the network. P21 is a downstream effector of p53 that activates the senescent program. Macip et al. found that decreasing levels of ROS, which induces p21, rescued cells from p21-induced senescence (30). Wang et al., 2011 focused into targeting survivin protein, a known substrate of Cdc2/Cdk1 kinase that promotes escape from the proliferative-arrest (31). In addition, recent exciting studies showed that silencing Ccna2 (antagonist of p21^WAF1/CIP1^), which is regulated by miR-29 and miR-124 miRNAs (p53 responsive miRNAs), triggered cellular senescence while its overexpression delayed it and improved cell viability (32). In fact, this mechanism of action was independent of the p53/p21^WAF1/CIP1^ axis, as it was also found in p21^WAF1/CIP1^ deficient cells (33).

Another scenario in which the “irreversible” concept has been challenged is related to the senescence induced in tumor cells. Saleh et al. described cellular senescence in tumor cells as an avenue to evade the cytotoxic impact of cancer therapy, allowing cells to extend their survival in a “dormant state” but retaining their self-renewal capacity and contributing to disease recurrence (34). It has been shown that oncogene-induced senescence (OIS) constitutes a mechanism to restrain cancer progression, in part by causing telomere dysfunction. Patel et al. showed that cells arrested by OIS can escape senescence by derepression of hTERT (human telomerase reverse transcriptase gene) expression; they both retained functional DNA damage responses and displayed high oncogene expression levels (35). In some human cancer cells and mouse models, p21^WAF1/CIP1^ found in the cytoplasm can exert oncogenic activities and inhibit proapoptotic proteins. This scenario is found in a p53 loss of function environment, promoting replication stress and triggering genomic instability to promote tumor aggressiveness. This agrees with findings of Milanovic et al. who found that the senescence-associated stemness in cancer cells is detrimental and confers high aggressive growth potential upon escape from cell cycle arrest (36).

Other examples related to “escape/reverse cellular senescence” are found in the reprograming of induced pluripotent stem cells (IPSCs). The use of Yamanaka factors (37) in combination with SV40 large T antigen (SV40LT) and/or hTERT are involved in senescence control (32). Once senescence is disabled, cells are more susceptible to either oncogenic transformation or reprograming (38). Indeed, Lapasset et al. demonstrated that a six-transcription factor combination generated IPSCs efficiently from senescent fibroblasts (39). Their combination could reset telomere size, gene expression profiles, oxidative stress and mitochondrial metabolism. Therefore, they could use senescent-derived IPSCs, redifferentiate them into fully rejuvenated cells, and erase cellular marks of aging. Studies demonstrated that during reprograming, some senescence-related pathways are affected. It was shown that, there is a positive correlation between senescence and reprogramming (40). *In vitro* and *in vivo*, increased expression of OCT4, SOX2, KLF4, and cMYC (OSKN) triggers reprogramming, but also leads to accumulated DNA damage resulting in some cells entering senescence. They demonstrated that tissues lacking p16^INK4a^/ARF do not undergo senescence but they also lack reprograming ability. By pharmacological manipulation of cellular senescence, using palbociclib (mimics p16^INK4a^) to increase senescence or navitoclax (proapoptotic drug) to reduce it, reprograming was increased or decreased respectively *in vivo*, showing the direct correlation between reprograming and cellular senescence. In fact, they identified one of the SASP factors, interleukin-6 (IL-6), as a key factor secreted by senescent cells promoting reprogramming. It was suggested that the same process occurs in conditions of injury or aging as well as in physiological conditions to promote tissue repair by inducing cell dedifferentiation. Other studies found SASP factors contribute to the expression of stem cells signature in mouse primary keratinocytes and *in vivo* in the liver, but that prolonged exposure to the SASP promoted cell cycle arrest (41).

What is perhaps surprising is that there is variation in the senescence characteristics, the timing of its development, and its SASP factors depending on the inducer and the cell type studied (22). Such variation can be partially understood by considering senescence as a cascade of events through which the cell passes before entering permanent cell arrest. In the DeCecco study even-though the timing differed with the stress inducer, senescence was a process initiated with p21 activity (early stages), with a changing pattern of SASP factors and finally with increased p16 activation which maintained senescence. Perhaps at early stages of the cascade, senescence is reversible while at later stages, there is permanence.

**Figure 2:**
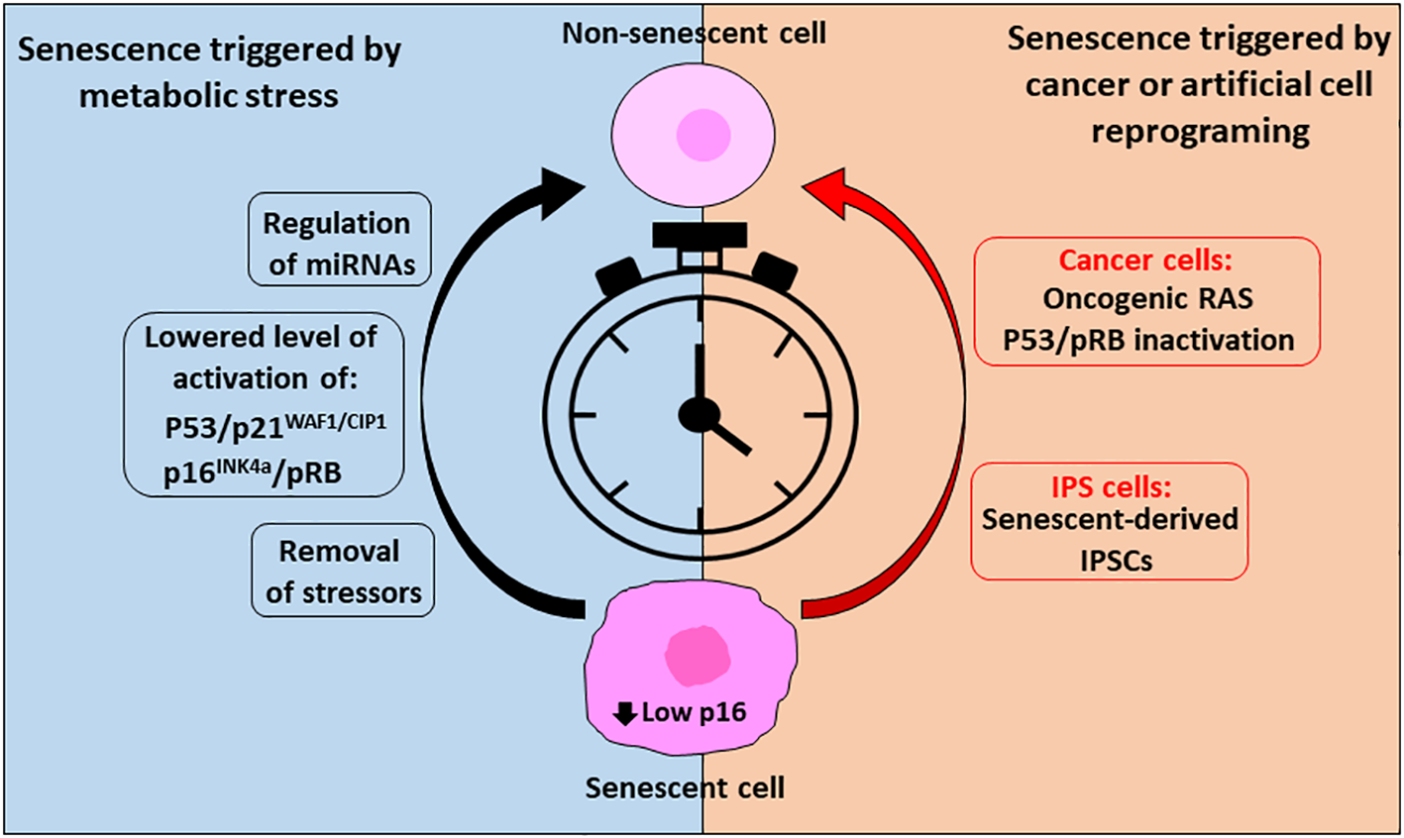
Reverse cellular senescent is possible, and timing is crucial. To reverse senescence triggered by metabolic stress or in cancer cells or cells triggered artificially through reprogramming (IPS cells) timing is crucial and success is dependent on low levels of p16 expression. In case of metabolic conditions inducing senescence, removal of the stressors followed by a recovery period can allow resumption of the cell cycle. In addition, lowered activation of the senescent effector pathways p53/p21^WAF1/CIP1^ and p16^INK4a^/pRB by regulation of their upstream and downstream effectors and/or by miRNAs can allow their escape from the senescent state. On the other side, cancer cells enter in senescence as a defense mechanism to survive through treatments of the disease, but they can exit this state and, in some cases, acquire an aggressive tumor behavior through expression of oncogenic RAS, regulation of pRB and upon p53 inactivation. In addition, reprograming of cells (IPSCs) can affect senescent pathways. However, it is possible to reprogram senescent cells into rejuvenated cells, through a precise combination of transcription factors.

In conclusion, it seems that under certain circumstances of senescence-cell states, cells can escape from induced cellular senescence before it becomes chronic and irreversible (**Figure 2**). Furthermore, it was observed that cancer cells use senescence as a dormant state to resist therapies and later escape and resume proliferation, conferring a very aggressive proliferation phenotype. Thus, reprograming cells to create IPSCs can affect senescence-related gene networks with deleterious consequences or alternatively give the potential to “rejuvenate” tissues.

## Cellular senescence in diabetes

Diabetes is a global pandemic that increases every year (42). Besides the different etiology of type 1 and type 2 diabetes, pancreatic beta cells have reduced or impaired insulin secretion in both and have reduced capability to compensate for the insulin demand to maintain normoglycemia. Although the underlying process causing insulin resistant is still not clarified, deficiencies or genetic polymorphisms of tyrosine phosphorylation of the insulin receptor, abnormalities of GLUT-4 receptor or dysregulations in the IRS proteins (43) have been suggested. Yet obesity, metabolic stress, endoplasmic reticulum stress, autoimmune attack (in case of Type 1 diabetes) and aging all can contribute.

In the last decade, several studies identified accumulation of senescent cells in important metabolic tissues and correlated their increase with development of the disease or its worsening (44). The number of β-galactosidase-positive adipocytes increases in diabetes. Furthermore, upregulation of p53 in adipocytes and macrophages promoted senescence and increased insulin resistance and inflammatory cytokines in mice (45). Most senescent cells have been found in white adipose tissues (WAT) (46–48). Transplantation studies of WAT from progeria or obese mouse models caused insulin resistance in host/recipient mice, demonstrating that senescent cells from fat are responsible, in part, to the onset of diabetes (45). Accumulation of senescent cells in fat tissue promoted inhibition of adipogenesis. This was mediated by activin A secreted by senescent fat progenitors, and inhibition of activin A rescued the impaired adipogenesis (49).

The liver is another metabolic organ in which senescence was found to participate in the development of its pathologies or in worsening them. Primary hepatocytes from patients with cirrhosis had significantly shortened telomeres compared to non-cirrhotic human samples. Telomere shortening correlated with expression of the cellular senescence marker β-galactosidase and was restricted to hepatocytes and was not observed in other cells in the liver, such as lymphocytes or stellate cells in areas of fibrosis (17). Another group found senescent activated hepatic stellate cells may be responsible for modulating the wound healing process by switching from a fibrogenic phenotype to an inflammatory phenotype (50). In addition, in non-alcoholic fatty liver disease in aging and obese populations, hepatocytes accumulate markers of cellular senescence (15). In the latter study the selective removal of senescent cells in INK-ATTAC mice (inducible whole-body transgenic mouse model that allows specific deletion of p16^Ink4a^ expressing cells after administration of a B/B homodimerizer that promotes activation of caspase 8 only in p16^Ink4a^ positive cells) (51) reduced hepatic steatosis in aging, obese, and diabetic mice. Furthermore, specific induction of senescence in hepatocytes by inactivation of the DNA repair gene *Xpg* caused liver steatosis. Interestingly, they corroborated that dietary restriction contributes to a healthier state and protects against hepatocyte senescence. Their conclusion was that removal of senescent hepatocytes, either by genetic manipulation or by the use of senolytics, such as the combination of desatinib (drug commonly used to treat chronic myeloid leukemia that induces apoptosis in senescent cells by inhibiting SRC tyrosine kinase) +quercetin (a flavonoid with antioxidant and anti-inflammatory effects that induces apoptosis of senescent cells by inhibiting the anti-apoptotic Bcl-xL protein) (D+Q), significantly reduced liver fat accumulation. In addition, they demonstrated a correlation between hepatocyte senescence and the severity of NAFLD (nonalcoholic fatty liver disease). They concluded that hepatocyte senescence is a major driver of fat accumulation in liver, potentially by mitochondrial dysfunction and impaired lipid metabolism. Such findings in different hepatic diseases suggest potential new effective treatments focused on removal of senescent cells and restoration of the functional hepatocytes.

Interestingly, these authors also reported that obesity induced by high fat diet or leptin deficiency increased anxiety and impaired neurogenesis in mice and that clearance of senescent cells from areas of brain implicated in regulation of food-control reduced the anxiety and restored the impaired neurogenesis (44). They found that cells in different brain areas did not have the same susceptibility to become senescent; high-fat diet feeding did not promoted accumulation of senescent cells in cortex, cerebellum or hippocampus. In addition, it has been found that cellular senescence occurring in aging is related to changes in the microbiome that can directly affect cognitive decline observed in neurodegenerative diseases, such as Alzheimer’s disease (52–56).

Another tissue that shows an impaired response to insulin signaling in scenarios of insulin resistance and diabetes is muscle (57, 58). It is important to take into account that, muscle is a heterogeneous tissue with different muscle fibers and cells (59, 60). Therefore, glucose metabolism might be different among them (59) and cellular senescence promoted by insulin-resistance or diabetes might not affect all muscle cell types to the same extent and such differences could be important in designing future treatments. It has been shown that diabetes impairs activation of satellite cells, the primary stem cells in adult skeletal muscle, resulting in both muscle atrophy (61, 62) and reduced oxidative capacity in skeletal muscle (63) that leads to muscle weakness and exhaustion (62, 64). Activation of the senescent pathway p53/p21^WAF1/CIP1^ promoted atrophy in muscle from older subjects (65). It is also known that in aging, muscle cells accumulate dysfunctional mitochondria (66, 67). Therefore, it is possible that cells in muscle undergo special type of senescence, which directly correlates to the impairment in mitochondrial function. MiDAS (Mitochondrial dysfunction-associated senescence), a special type of senescence which is a consequence of dysfunctional mitochondria, differs from other types. It is characterized by a decreased NAD^+^/NADH ratio mediated by AMPK, which promotes activation of p53 and subsequent NF-kB inhibition and results in lack of expression of IL-1 dependent factors in their SASP (18). In the same study they identified sirtuins (SIRTs) as key factors in MiDAS. As previously suggested, for future treatments for sarcopenia and aging skeletal muscle, the crosstalk between the different cell types in the muscle needs to be taken into account. In addition, attention should be taken to the senescence on muscle stem cells and the induction of potential reprograming.

Additionally, the contribution of senescent pancreatic beta cells to development of both Type 1 and 2 diabetes and the subsequent improvement of glucose homeostasis with the removal of those senescent cells was reported in 2019 (14, 16). Together these discoveries across various metabolic tissues suggest that new potential treatments of preventing, reversing or removing senescent cells will have metabolic benefit.

## Main contributor to development and worsening of diabetes: senescent pancreatic beta cells

Aging plays an important role in the appearance and accumulation of senescent pancreatic beta cells. Besides the natural/chronological aging of the organism, different pathological conditions can accelerate the appearance of dysfunctional and senescent pancreatic beta cells, promoting an early aging of the organ.

In 2017, Aguayo-Mazzucato et al. identified markers of aging beta cells and showed heterogeneity in how the beta cell population aged (68). Using the senescence marker of increased β-galactosidase activity, even young mice had a significant population of senescent beta cells and this population increased with age. The correlation of increased senescent beta cells and diabetes was then shown in Type 1 diabetes by Thompson et al. (16), and for Type 2 diabetes by Aguayo-Mazzucato et al. (14). In non-obese diabetic (NOD) mouse model, a population of senescent pancreatic beta cells that secreted SASP factors contributing to propagation of the immune-mediated destruction process was identified. Interestingly, senescent beta cells accumulated prior to onset of Type 1 diabetes, seen as increased of CDKN1A, IL-6 and SERPINE-1 in autoantibody-positive donors as compared to non-diabetic control donors (16). Since they found upregulated expression of antiapoptotic gene Bcl-2 in these senescent pancreatic beta cells (as previously described to occur in senescent cells (69), they used senolytic small molecules against Bcl2 to target the senescent cells and clear the islets of those dysfunctional cells; in doing so they prevented the onset of diabetes. About the same time, Aguayo-Mazzucato et al. (14), generated a “signature” to identify senescent beta cells and showed that senescent beta cells had decreased expression of identity genes and expressed many of the “disallowed” genes normally not expressed in functional beta cells, a phenotype that contributed to worsening of the metabolic profile. Importantly they showed acute (with the insulin receptor antagonist S961) or chronic (high fat diet) insulin resistance accelerated the accumulation of senescent beta cells. They saw improved glucose homeostasis when they deleted the senescent beta cells in INK-ATTAC mice whether aged, fed with high-fat diet, or treated with S961. Similarly, oral treatment with the senolytic ABT263 (also known as Navitoclax, induces apoptosis in senescent cells by targeting BCL2/BCL-xL/BCL-W) improved the glucose metabolism and provided partial restoration of the beta cell identity. Importantly they show that senescent beta cells increased in human islets with age, BMI and with Type 2 diabetes.

In a follow-up study (19) they defined a specific beta cell SASP signature based on proteomic analysis from conditioned media from senescent mouse and human primary islets as well as murine beta cell line MIN6 cells. They found differing secretomes using primary β-gal positive beta cells senescent from *in vivo* environment and age and MIN6 cells treated with bleomycin (inducer of DNA damage). SASP in MIN6 cells correlated with *Cdkn1a* expression, corresponding to early senescence. To test if the senescence seen in the beta cells in response to acute insulin resistance were also early stage and reversible, they compared single cell RNA seq data from murine islets after two weeks of treatment with insulin receptor antagonist S961 to accelerate beta cell senescence, islets from mice that then had two weeks of recovery (normal glycemia and plasma insulin levels) and control (saline treated) mice. While analysis of the beta cells showed most of the S961-treated beta cells had changed gene expression with loss of identity genes, those from the recovery animals had mostly returned to control expression levels. The gene expression significantly correlated with *Cdkn1a* expression (p21), therefore suggesting that early senescence could be reversed when the inducer of cellular senescence was removed. One might speculate that in humans, reversal of the earlier stages of senescence may be the basis of the remission from type 2 diabetes seen with intensive treatment with insulin or gastric bypass (70–76).

The insulin pathway is being studied as a potential target to prevent cellular senescence in metabolic tissues. Human hepatocytes exposed to prolonged hyperinsulinemia increased levels of p53 and p21 and promoted cellular senescence (76). Besides the treatment with common senolytics, such as desatinib and quercetin, reducing the levels of insulin receptors blunted cellular senescence as well, as shown using the liver-specific insulin receptor knockout mouse model (LIRKO) (77). In addition, IGF1 (insulin-like growth factor binding protein 1), can cross-activate with insulin receptor, and similar to insulin, promoted increase in p53 and senescent phenotype (76). In fact, it was previously shown in human dermal fibroblast and endothelial cells that IGF5 can induce cellular senescence through a p53-dependent mechanism. IGFBP5 has been suggested as a potential target for vascular diseases since atherosclerotic arteries showed strong staining for IGFBP5 (75). Another study (74) showed a dual effect of IGF-1 in human primary fibroblast (IMR90 cells) and mouse embryonic fibroblast (MEFs): acute exposure promoted cell proliferation but prolonged exposure induced premature senescence by modulation of activation of p53 through SIRT-1. In pancreatic β cells, IGF1R signaling was reported as key to aging-induced β-cell dysfuntion (68). Decreased IGF1R contributed to attenuated senescence in pancreatic beta cells and improved their function (78).

Therefore, in pancreatic beta cells as in other metabolic tissues that coordinate the systemic response to metabolic stress, the modulation or elimination of senescent cells can blunt progression of diabetes and ameliorate the symptoms. Moreover, their SASP profile may provide information of the senescent stage of the cells in order to tailor treatments to the early- or late- senescence.

## Senolytics and senomorphics to erase senescent pancreatic beta cells

**Figure 3:**
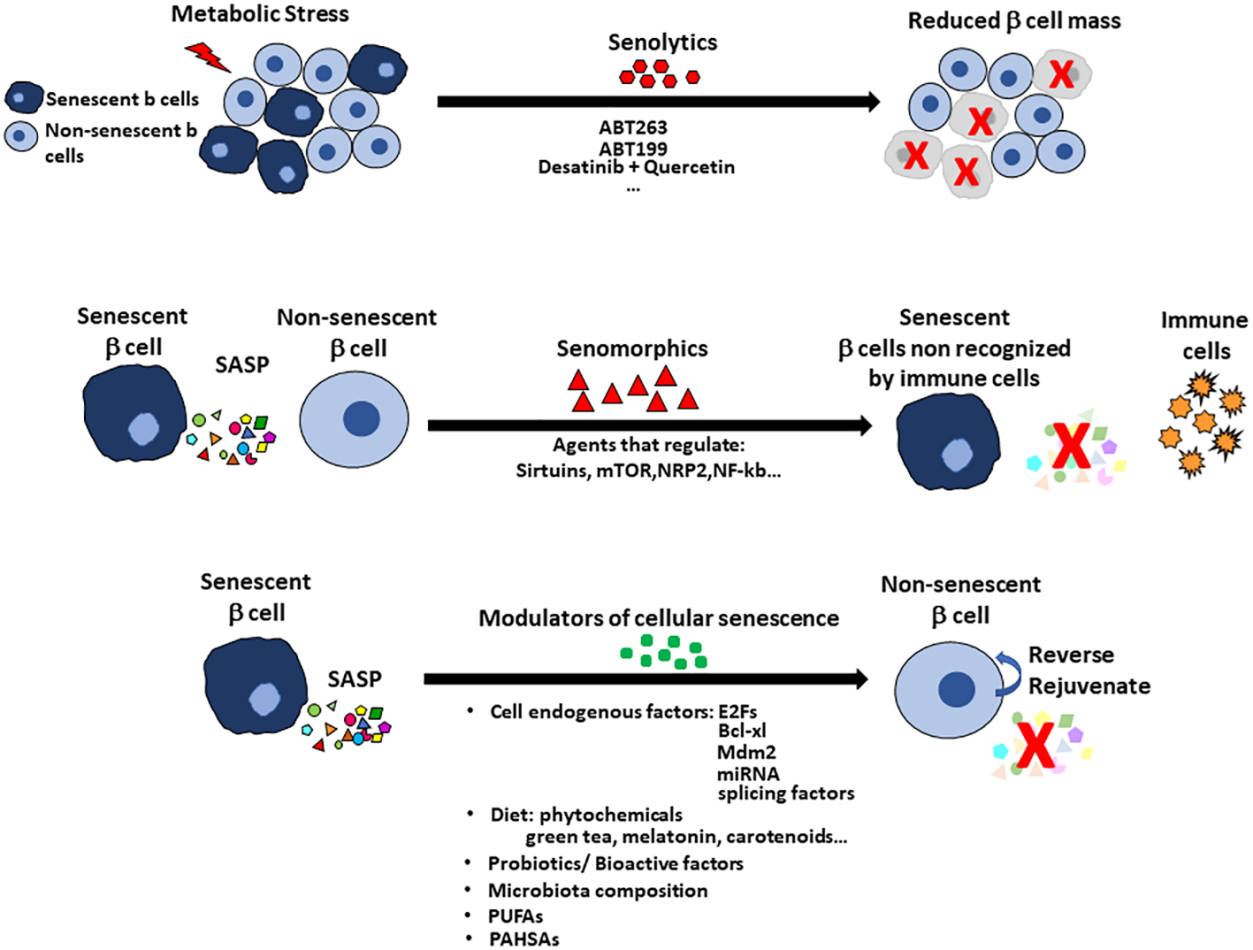
Treatments to alter cellular senescence include senolytics, senomorphics and modulators of cellular senescence (MCS) with pancreatic islets as an example. Senolytics target the increased anti-apoptotic pathways so that the cells can be cleared. The disadvantage of senolytics is the cell population in the specific tissue is reduced, which increases the problem of reduced beta cell mass in the diabetic pancreas. Senomorphics modulate or reduce the expression of SASP without induction of apoptosis. Their use has the disadvantage that the senescent cell remains in the tissue and so treatment may need to be continuous. Senomorphics usually target sirtuins, mTOR factors, NRP2 or NF-kb pathway. Senolytics and senomorphics lack tissue specificity but new therapies using nanoparticles for targeting specific cells are being tested. Modulators of cellular senescence (MCS) offer the advantage to preserving the beta cell mass by reversing the senescent state and rejuvenating the tissue. Modulators of cellular senescence include factors intrinsic to the cells (eg E2Fs, Bcl-xl, Mdm2, miRNAs, splicing factors) and factors found in the diet (eg probiotics, bioactive factors and even perhaps specific members of the gut microbiota. Omega-3 fatty acids and PUFAs have positive effects on cellular senescence with recent report of PAHSAs preventing and rejuvenating beta cells.

Cellular senescence has been named “a major contributor” to the onset of diabetes, so learning how to selectively target the senescent cells is a very active research area. Yet cellular senescence has many aspects. It can be beneficial, constituting a tightly regulated physiological process for development and tissue repair. The recognition of SASP factors by the immune system works and the cells are targeted and removed (2, 6, 79, 80). However, with age the immune clearance may become impaired so senescent cells can accumulate with detrimental consequences (14, 16, 81–84). Based on that, research for modulators of cellular senescence have taken two paths: 1) a search for molecules that promote the senescent state of the cells for treatment of cancer, 2) to find compounds that suppress the senescent state or remove the senescent cells to rejuvenate the remaining tissue. Of the latter there are two types, senolytic and senomorphic (**Figure 3**).

Senescent cells have elevated antiapoptotic pathways that allow them to survive. Senolytic drugs are selected for their targeting of these antiapoptotic pathways and so selectively deleting the senescent cells. Many have orally administration and approval by the US Food and Drug administration (FDA). The effectiveness of senolytics, as well as senomorphics, has been studied *in vitro* with murine and human cells and *in vivo* in experimental animals.

In the setting of type 2 Diabetes, quercetin alone or in combination with dasatinib + quercetin (D+Q) has been used to target the HIF1α pathway, which is significantly upregulated in β-galactosidase positive beta cells, and ABT263 for targeting the Bcl2 pathway. Combination of D+Q effectively removed β−Gal positive beta cells from mice treated with S961 and improved blood glucose levels. Treatment with ABT263 improved glucose metabolism, beta cell identity gene expression and selectively reduced p16^Ink4a^ in islets from treated animals (14). In type 1 diabetes, BH3 mimetics (compounds that mimic the binding of the BH3 to the prosurvival factors in the BCL2 antiapoptotic pathway, such as ABT-199 (also known as venetoclax, targets only BCL2) were effective in NOD mice. Interestingly, in humans, senescent beta cells accumulated prior to onset of Type 1 diabetes as seen by the increased expression of CDKN1A (p21), IL-6 and SERPINE-1 in pancreas from autoantibody-positive donors as compared to non-diabetic control donors (16). Thus, senolytic treatment at early stages may be able to halt or delay the onset of type 1 diabetes in humans as well.

While there may be benefits by deletion of the senescent cells, senolytics would reduce the mass of beta cells which is already reduced in early Type 1 or Type 2 diabetes (**Figure 3**). In contrast to senolytics, senomorphics target SASP-related pathways (Sirtuins, mTOR and transcription factors such as NRF2 and NF-kB) and block SASP secretion but do not kill the cells. The senomorphic effects of metformin, a common treatment for type 2 diabetes, have been studied in adipose tissue and bone marrow mesenchymal stem cells (85–87). Metformin lowers glucose levels by inhibiting hepatic gluconeogenesis, so its effect on senescence may result solely from its effective lowering of blood glucose levels that prevents DNA damage and inflammation that are key triggers of cellular senescence.

Additionally, investigators are searching for other treatments against cellular senescence. One approach is to target senescent cells using a sophisticated method of galacto-oligosaccharide-coated nanoparticles, based in the increased lysosomal mass and β-galactosidase activity of senescent cells (88). Another approach leverages the high lysosomal activity of the senescent cells by administrating ATPase inhibitors that promote the rupture of lysosomal vesicles, deregulating the glutamine metabolism and prompting apoptosis (89).

## Modulators of cellular senescence: promising treatment for senescent cells

Another approach is searching for molecules that can modulate cellular senescence existence in tissues. Modulators of cellular senescence (MCS) can act at different levels. Some MCS target pathways that evade cell cycle arrest, such as the inhibitor of mTOR pathway rapamycin, shown to reverse cellular senescence in embryonic fibroblasts (90). Rapamycin stabilizes Nrf2 to delay cell cycle arrest, and this effect seems to be independent on mTOR activation (91, 92). Rapamycin can also act as a senomorphic by suppressing SASP through its inhibition of mTOR. Even so rapamycin is also a key regulator of cellular senescence and lifespan through mTOR and its control of nutrient sensing (1, 93–96).

Transcription factors can also act as MCS. E2F1 plays an essential role in regulation of the cell cycle progression and is a main target for the tumor suppressor pRB. In human cancer cells the knockdown of E2F1 transcription factor induced replicative senescence and stable cell cycle arrest whereas its overexpression made the cells resistant to induced senescence (97). The down-regulation of E2F1 found in oncogenic senescence seemed independent of the integrity of pRb and p53.

The anti-apoptotic protein Bcl-xl that serves as target for senolytic drugs (e.g., ABT737 or ABT263) is also considered a modulator of senescence. During earlier stages of senescence induction, Bcl-xl helps mitochondria cope with the high metabolic demand to secrete the SASP needed to recruit the immune system for clearance of the senescent cells. However, as senescence become more established, senescent cells acquire a dysfunctional mitochondrial phenotype; Bcl-xl expression prevents the accumulation of these impaired mitochondria and their detrimental effect on tissue homeostasis. In addition, Bcl-xl contributes to preserve immunosurvillance of senescent cells (98, 99).

Another class of modulators of cellular senescence are micro-RNAs. Analysis of microarrays of miRNA and mRNA from kidneys of aged (20-months old) mice compared to young (2-month-old) revealed the upregulation of several p53-responsive miRNAs, including miR124, miR34a/b/c and miR-29a/b/c (33, 100–102). Overexpression of miR124, miR34a and miR29a in murine embryonic fibroblasts (MEFs) increased β-galactosidase positive staining and increased p16 expression, confirming their role in promoting cellular senescence. Cyclin A2 (Ccna2), a common target for several p53-responsive miRNAs (miR124 and miR129), acts as an antagonist of p21 in cell cycle regulation, and its silencing contributes to triggering cellular senescence. In contrast, overexpression of Ccna2 delayed cellular senescence and reversed the senescence induced by miR124 and miR129. The p53/miRNAs/Ccna2 pathway seems to act as a modulator of cellular senescence independent of p53/p21 pathway, since p53-responsive miRNAs are elevated in senescence in p21-deficient cells. In addition, it has been studied that miRNAs, exert their role of modulators of cellular senescence by having effects in other key regulators of cellular senescence as p21^WAF1/CIP1^ (103) or p16^INK4a^ (104–106), which confers modulation at different levels of the senescent pathways.

With aging there are changes in genes that control alternative splicing that acts as a major regulator of gene expression and provides genomic plasticity (107, 108). Modulation of splicing factor levels can reverse cellular senescence in human primary fibroblasts (109). Resveratrol is reported to extend lifespan in several organism through activation of SIRT1, an NAD-dependent protein deacetylase (110). Treatment of human fibroblasts with analogues of resveratrol promoted shifts in expression patterns of multiple splicing factors characteristic of early passage cells and reduced biomarkers of cellular senescence. Elevated splicing factor expression is associated with telomere elongation, and in growth permissive conditions, previously senescent population can proliferate, measured by increase in ki67 staining, which indicates re-entry in the cell cycle (109). Thus, splicing factors constitute potential targets to modulate cellular senescence.

The search of modulators of cellular senescence within the diet is getting more attention. It has been shown that differences in dietary exposures provided different levels of protection against pathogen infections and development of diseases (111–113). In this regard, dietary bioactive factors have been found to be important to modulate the senescence of immune cells (immunosenescence), which indirectly affects to cellular senescence in other tissues/cells. This area comprises the study of phytochemicals with cytoprotective effects and immunomodulator potential (plant-based metabolites such as polyphenols, alkaloids, carotenoids, etc.) (114). Consumption of green tea, melatonin or carotenoids showed effects in against immunosenescence, and in some cases reversed the SASP-induced increase of beta-galactosidase and expression of cell cycle inhibitory genes in mouse macrophages (115). In addition, consumption of certain vitamins and minerals had positive effects in reinforcing immune cell population (116, 117).

The study of probiotics as another source of cellular senescence modulators is a growing area of research. It has been demonstrated that the gut microbiota modifies and regulates the immune response. Known “good bacteria”, such as *Lactobacillus case*i or *plantarum*, have the potential to modulate oxidative stress, innate immunity, immunosenescence and even increase lifespan of *Caenorhabditis elegans* (115, 118). Other strains of bacteria such as enterococcus in combination with some types of lactobacilli had positive effects in the expression of tight junction proteins of the intestine maintaining its barrier function (119).

The effects of bioactive factors and probiotics constitute an interesting field to study modulation of cellular senescence at the systemic level (4, 120, 121) and, in particular, to the development of diabetes. The presence and abundance of certain types of bacteria in the gut microbiome has been positively or negatively correlated to Type 2 diabetes (122). In the same context, gut microbiota has a key role in early beta cell development and proliferation (123). As well, several studies provided evidence of regulation of insulin sensitivity by the gut microbiome (124–126). Furthermore, fecal transplantation has been reported to affect beta cell function in the recipient (127–130). Thus, targeting the gut microbiota is a growing area to search for treatments to prevent activation of the senescence program in pancreatic beta cells.

Another field for further search for modulators of cellular senescence is related to fatty acids, particularly the polyunsaturated fatty acids (PUFAs). Murine studies administering PUFAs, showed attenuation of several markers of age-related immunosenescence (described as the lower precision and activity of the adaptative immune system), inflammaging (indicating an increased pro-inflammatory activity of the innate immune system) and Th1/Th2 cytokine imbalance (131, 132). PUFAs are present in different foods, and many are FDA approved. Several clinical trials showed the benefits of oral administration of these compounds with regard to cellular senescence. Administration of n-3 PUFAs, such as eicosapentaenoic acid (EPA) and docohexaenoid acid (DHA), was associated with increased telomere length in blood leucocytes and resulted in increased proliferative response and attenuation of several markers of immunosenescence (133). Interestingly, in a population from China, individuals having higher n6:n3 PUFA ratio in plasma and lower EPA and DHA (n3 PUFAs) correlated with shorter leucocyte telomere length and increased coronary artery disease (134). Additionally, supplementation with EPA and DHA improved cognitive impairment in an elderly population (135). However, none of these clinical trial studies reported effects of PUFAs on pancreatic beta cells.

Our own studies recently reported Palmitic acid hydroxy stearic acids (PAHSAs) as novel endogenous modulators of cellular senescence, with the potential to prevent and reverse cellular senescence in metabolically stressed, pancreatic beta cells (20). PAHSAs constitute a subfamily from the signaling lipids called Fatty Acid esters of Hydroxy Fatty Acids (FAHFAs), with anti-inflammatory and anti-diabetic effects (136). Using prediabetic NOD mice chronically treated with PAHSAs by oral gavage and *in vitro* models for metabolic stress in MIN6 cells and in human islets, we showed that PAHSAs directly prevented, as well as reversed, expression of genes and proteins related to cellular senescence in pancreatic beta cells. The mechanism by which PAHSAs exert their effects on beta cells is by acting on p53’s negative regulator Mdm2 and other factors that control the p53/p21 pathway, as well as in the above mentioned E2F1 factors. PAHSAs exerted a modulatory effect preventing activation of p53 and the cascade of downstream effectors that induce senescence. PAHSAs treatment in NOD mice resulted in significantly decreased senescent proteins (p21, p16, Hmgb1 or γH2Ax) in beta cells as well as in alpha cells. In addition, a novel feature of treatment with these lipids was the enhanced expression of DNA-repair and protective (glutathione metabolism) genes, which in cellular senescence are down-regulated. Therefore, we believe that these lipids (PAHSAs) constitute a great target to generate potential therapies against cellular senescence in pancreatic beta cells.

Therefore, in the context of the detrimental effects of cellular senescence observed in pancreatic beta cells, the use of such modulators of cellular senescence, with the potential to reverse senescence markers and restore the dysfunctional beta cells would preserve the functional beta cell mass in contrast to senolytics.

## Conclusions and future perspectives

Since cellular senescence has been shown as the target to ameliorate or halt the progression of diabetes, efforts have focused on the effectiveness of senolytics and senomorphics. However, the co-lateral damages of senolytics push the need of finding new therapies that erase cellular senescence from pancreatic beta cells as well as the other involved metabolic tissues. More research on possible therapies that “rejuvenate” or “reverse” is needed, including compounds that target genes and proteins identified to be involved in development of cellular senescence.

## Author contributions

MFRC and SB-W generated the ideas and wrote the manuscript. All authors contributed to the article and approved the submitted version.
